# Personalized Management of Myocarditis and Inflammatory Cardiomyopathy in Clinical Practice

**DOI:** 10.3390/jpm12020183

**Published:** 2022-01-30

**Authors:** Agata Tymińska, Krzysztof Ozierański, Aleksandra Skwarek, Agnieszka Kapłon-Cieślicka, Anna Baritussio, Marcin Grabowski, Renzo Marcolongo, Alida LP Caforio

**Affiliations:** 1 First Department of Cardiology, Medical University of Warsaw, 1a Banacha St., 02-097 Warsaw, Poland; agata.tyminska@wum.edu.pl (A.T.); s073784@student.wum.edu.pl (A.S.); agnieszka.kaplon@gmail.com (A.K.-C.); marcin.grabowski@wum.edu.pl (M.G.); 2 Division of Cardiology, Department of Cardiac, Thoracic and Vascular Sciences and Public Health, University of Padova, 2-35128 Padova, Italy; anna.baritussio@aopd.veneto.it (A.B.); renzo.marcolongo@gmail.com (R.M.); alida.caforio@unipd.it (A.L.C.)

**Keywords:** anti-heart autoantibodies, endomyocardial biopsy, heart failure, individualized therapy, inflammation, immunosuppressive treatment, personalized medicine

## Abstract

Myocarditis is an inflammatory heart disease induced by infectious and non-infectious causes frequently triggering immune-mediated pathologic mechanisms leading to myocardial damage and dysfunction. In approximately half of the patients, acute myocarditis resolves spontaneously while in the remaining cases, it may evolve into serious complications including inflammatory cardiomyopathy, arrhythmias, death, or heart transplantation. Due to the large variability in clinical presentation, unpredictable course of the disease, and lack of established causative treatment, myocarditis represents a challenging diagnosis in modern cardiology. Moreover, an increase in the incidence of myocarditis and inflammatory cardiomyopathy has been observed in recent years. However, there is a growing potential of available non-invasive diagnostic methods (biomarkers, serum anti-heart autoantibodies (AHA), microRNAs, speckle tracking echocardiography, cardiac magnetic resonance T1 and T2 tissue mapping, positron emission tomography), which may refine the diagnostic workup and/or noninvasive follow-up. Personalized management should include the use of endomyocardial biopsy and AHA, which may allow the etiopathogenetic subsets of myocarditis (infectious, non-infectious, and/or immune-mediated) to be distinguished and implementation of disease-specific therapies. In this review, we summarize current knowledge on myocarditis and inflammatory cardiomyopathy, and outline some practical diagnostic, therapeutic, and follow-up algorithms to facilitate comprehensive individualized management of these patients.

## 1. Background

Contemporary heart failure (HF) management requires consideration of the many factors that might influence an individual’s response to treatment, particularly the disease etiology. This is especially true in patients with myocarditis/inflammatory cardiomyopathy, where standard HF medications do not influence the underlying cause of the disease. Due to the large variability in its clinical presentation, and unpredictable course, myocarditis is still considered an orphan disease with a challenging diagnosis. However, myocarditis is not a rare disease. The incidence of first-time hospitalizations with suspected myocarditis was reported at approximately 60 per 100,000 people per year in a recent 10-year observational study [1]. Moreover, current reports show that the incidence of myocarditis and inflammatory cardiomyopathy has been rising in recent years/decades [1,2].

Diagnosis of myocarditis should follow a systematic approach (with a common definition following strict criteria and with a final confirmation by endomyocardial biopsy (EMB)), while in most situations, it is still a clinician judgment-based diagnosis of exclusion. This was highlighted in a recent Polish nationwide study, which showed that performance of the recommended diagnostic tests (in particular, EMB and cardiac magnetic resonance (CMR)) was very low in clinical practice [1]. The complications of sub-optimally treated myocarditis may be serious, including reduced quality of life and ability to work, arrhythmias, dilated cardiomyopathy, death, or heart transplantation.

Still, substantial work remains in order to personalize management in this sub-group of HF patients to maximize the benefit of pharmacologic interventions while limiting adverse outcomes. In this review, we aimed to summarize current knowledge, and outline possible, crucial elements of the comprehensive, individualized management of myocarditis and inflammatory cardiomyopathy.

## 2. Definitions

To provide optimal treatment of myocarditis, it is necessary to obtain an adequate diagnosis and identify its etiology. It should be highlighted that the diagnosis of myocarditis is only established following histological, immunohistochemical, and molecular confirmation based on EMB. Therefore, the term “myocarditis” should refer only to EMB or autopsy-proven diagnosis according to the European Society of Cardiology (ESC) criteria [3]. The recent coronavirus pandemic outlined that failure to apply a standardized diagnosis leads to misdiagnosis and information noise [4]. The key definitions required for a better understanding of myocarditis and making an adequate diagnosis are presented in Table 1.

## 3. Emerging Etiologies and Pathophysiology of Myocarditis

**Figure 1:**
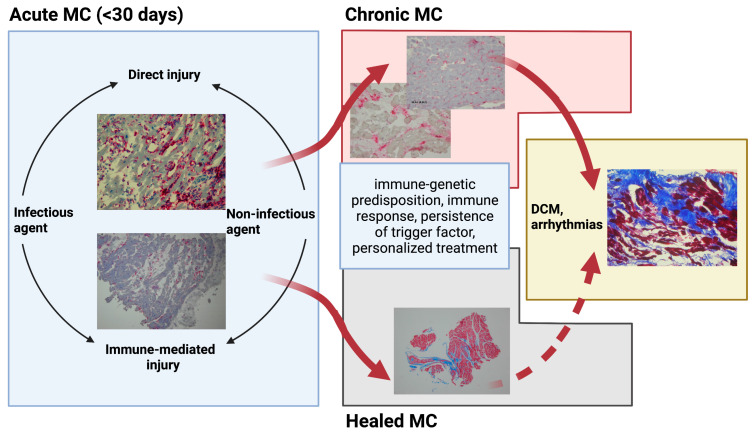
Pathogenesis and natural course of myocarditis. DCM: dilated cardiomyopathy; MC: myocarditis. Created with BioRender.com (accessed on 4 January 2022).

Myocarditis is a multi-factorial condition, with an inflammatory reaction as the main manifestation of the underlying pathological agent–host interaction. Inflammatory infiltration of the myocardium irrespective of the etiological factor leads to temporal or definitive compromised cardiac function. On top of the occurrence of the inflammatory process lies immune-genetic predisposition, which seems to determine the disease origin and course [3,6,7]. A schematic pathogenesis and natural course of myocarditis is presented in Figure 1. 

Numerous etiological factors trigger the innate immune response and complex pathophysiological mechanisms maintaining and driving the cascade of inflammation [3,5,8]. From the clinical point of view, two general etiological pathways may be distinguished: infectious and non-infectious. Finally, in most non-infectious cases, but also possibly in infectious cases, myocarditis is based on immune-mediated mechanisms. The diagnosis of a specific etiology (infectious vs. non-infectious) and confirmation of the inflammation determines the treatment and prognosis of a patient. 

Among infectious agents, viruses are most frequently identified in EMB samples (in approximately 15–30%), although more frequently if EMB is performed in the early stage of acute myocarditis [9,10]. Bacteria, fungi, parasites, etc. are less commonly found. However, one needs to differentiate among the viruses those that infiltrate myocardial tissue (cardiotropic viruses), hence directly leading to myocardial damage and inflammation, and those that induce myocardial damage without invading the myocardium itself, i.e., through cytokine storm or cell/antigen-mediated immune reaction [8]. Currently, the predominant cardiotropic viruses associated with myocarditis and inflammatory cardiomyopathy are Parvovirus B19, and Herpesviridae family (herpesvirus 6 (HHV6); and Coxsackie virus, Echovirus, Epstein–Barr virus (EBV), Cytomegalovirus, hepatitis C virus (HCV), and influenza A and B virus are less frequent. Severe Acute Respiratory Syndrome Coronavirus 2 (SARS-CoV-2) has recently been proposed to be associated with myocarditis; however, there is still no evidence that SARS-CoV-2 is able to invade and/or directly damage cardiomyocytes [4].

The primary cardiotropic viruses are of great interest, because of their directly detectable effect at the level of cardiomyocytes. They have been proven to attach to the surface of cardiomyocytes through the coxsackievirus-adenovirus receptor (CAR) [11], replicate in host cells, cause disruption of cardiomyocytes’ cytoskeletal proteins [12], and consequently, myocardial injury. The endotheliotropic viruses include Parvovirus B19, which can persist in the heart with the possibility of reactivation episodes [8,13]. Viruses from the Herpesviridae family are lymphotropic and reside in cells of the immune system, leading to myocardial injury [8]. On the other hand, viruses from the Coronaviridae family (i.e., SARS-CoV-2) might cause myocarditis indirectly (virus negative immune-mediated) by activating the various components of the immune system. SARS-CoV-2 infection might also trigger or accelerate already established subclinical autoimmune forms by the hyperinflammatory state [14].

Non-infectious causes of myocarditis include the direct toxic effect of a triggering factor (i.e., medications, alcohol, cocaine, etc.) or underlying systemic immune-mediated diseases (SIDs). 

The role of the immune system in the pathogenesis of myocarditis is crucial, and can be both beneficial and detrimental. Prompt and adequate immune reaction is indispensable for effective pathogen clearance. However, the same defense mechanisms, which are directed against the triggering factor, can lead to the phenomenon of autoimmunity [3]. Myocarditis can occur as an isolated organ-specific autoimmune process or in the context of systemic immune-mediated diseases (SIDs) [15]. The underlying pathology of myocarditis during autoinflammatory diseases is not well documented. In principle, it might represent unprovoked myocardial inflammation in the absence of autoantibodies or autoreactive T lymphocytes. Myocarditis in the context of an autoimmune disease is caused by inadequate B, T, and dendritic cell responses, leading to the production of autoantibodies, which recognize self-antigens on the surface of cardiomyocytes and play a major pathogenetic role. Both mechanisms of immune-mediated myocarditis can form a continuum with mixed forms [14].

A recently observed large number of small reports describing myocarditis induced by immune-checkpoint inhibitors (ICIs), which are used in novel cancer therapies, indicate the possibility of new etiological factors emerging with the progress in medicine, and the need for frequent updates [16]. ICIs (e.g., ipilimumab, nivolumab, and pembrolizumab) have been approved for use in several types of cancer, i.e., non-small-cell lung cancer, small-cell lung cancer, and colorectal carcinoma [17]. The occurrence of ICI-induced myocarditis is around 1% according to a recently published multicenter observational registry [18]. This makes it an uncommon complication, which is, however, expected to be reported more often due to expanding indications for ICI use. It is characterized by an unusually high fatality rate of 30–50%, which can be attributed to both the fulminant course of myocarditis itself and the multimorbidity of cancer patients. ICI-induced myocarditis is very likely due to an induction of autoimmunity of the heart and other organs in susceptible individuals. Following EMB confirmation, it requires the interruption of therapy with ICI and treatment with steroids with or without other immunosuppressive agents [19]. It has been suggested that myocytes and tumor cells may share autoantigens, which in turn cause the immune system, stimulated by ICIs, to target both structures on the basis of a “molecular mimicry” mechanism, similar to the one described in the classical model of virus-induced myocarditis [19]. This is of particular interest for personalized medicine, as it may be indispensable in the future to identify the tumor antigens that may possibly cross-react in each patient prior to administering therapy. This type of myocarditis from the area of cardio-oncology, due to its interdisciplinary character, requires team-oriented individualized therapy.

Mass vaccination with an mRNA vaccine against SARS-CoV-2 (BNT162b2, Pfizer–BioNTech) in Israel has recently been reported to be a potential emerging cause of myocarditis [20]. This observation was based on 2 studies, which reported a frequency of myocarditis occurrence in individuals who had completed the anti-SARS-CoV-2 vaccination cycle of 136/5,000,000 [21] and 54/2,500,000 [22], respectively, which was higher compared to non-vaccinated individuals. In the described patient cohorts, the illness was most frequent in young males (16–19- [21] and 16–29-year-olds [22], respectively) and took a benign self-limiting course. In both studies, the diagnosis was clinician based, with low and very low application of CMR and EMB, respectively. 

Similarly, researchers from Denmark performed a population-based cohort study to analyze the rates of myocarditis or myopericarditis after mRNA vaccination. Out of the 3.5 million residents living in Denmark aged 12 years and older who received BNT162b2 and 500,000 people vaccinated with the mRNA-1273 (Moderna) vaccine, the incidence of myocarditis or myopericarditis within 28 days was 1.4 and 4.2 per 100,000 vaccinated individuals, respectively. This diagnosis was defined as a hospital diagnosis of myocarditis or pericarditis plus an increased troponin level and admission lasting >24 h (information on the frequency of the performed EMB or CMR was lacking). The rates in Denmark are lower than those reported by researchers in the United States and Israel. Surprisingly, in the BNT162b2 vaccine group, the outcome risk was significantly higher in females only. The authors concluded that vaccination with mRNA-1273 was associated with a significantly increased risk of myocarditis or myopericarditis in the Danish population. However, the overall risks are low and must be balanced against the individual and societal benefits associated with vaccination [23].

Due to this limitation of the presented studies, we can neither identify the etiological factor nor draw conclusions about the causative role of the vaccine. It is true that there is a temporal association between the administration of the vaccine and myocarditis, but myocarditis etiology is unspecified, and may represent a random association and/or naturally occurring viral or immune-mediated myocarditis that is accelerated or precipitated by the vaccine [20]. Considering the fact that myocarditis that is temporally associated with mRNA vaccines is very rare, refusal of the vaccine to young patients is discouraged. In case of myocarditis occurring after the vaccine administration, we recommend the diagnostic protocol provided by ESC encompassing the performance of EMB [3,20].

## 4. Clinical Presentation and Complications in Patients with Myocarditis

Patients with suspected myocarditis are mostly young (in a recent nationwide study, the median age was 32 and 46 years in males and females, respectively) and the majority are male (approximately 75%) regardless of the age group [1]. Seasonal changes in the incidence of suspected myocarditis have also been observed, with the highest rates of hospital admissions occurring from late autumn to early spring, which might reflect infectious and particularly viral causes [24]. The onset of myocarditis may be preceded (days to weeks) by a respiratory or gastrointestinal infection in up to 80% of cases [25]. 

The clinical manifestations of myocarditis range from a subclinical course with mild symptoms of chest discomfort and transient palpitations with no compromised myocardial function, to fulminant myocarditis with cardiogenic shock or life-threatening ventricular arrhythmia; acute, subacute, or chronic HF with or without a dilated cardiomyopathy phenotype; and a variable duration of preceding symptoms or in the peri-partum [8,26,27].

Current data indicate that biopsy-proven myocarditis resolves in a few weeks in approximately 50% of cases without complications, but about 25% develop persistent cardiac dysfunction and 12–25% may die or deteriorate to end-stage dilated cardiomyopathy [3,28]. Worse outcomes are observed in patients with fulminant presentation, left or biventricular dysfunction, advanced NYHA and HF presentation at diagnosis, and specific histotypes, in particular giant-cell, eosinophilic myocarditis, and cardiac sarcoidosis [3,26]. Biopsy-proven myocarditis with such high-risk features at diagnosis has a high mortality rate if not diagnosed and treated in time [26]. The 1-month mortality for fulminant myocarditis requiring an intensive care unit is greater than 40% [29] while the 4-year mortality in untreated giant-cell and eosinophilic myocarditis is extremely high, reaching 90% [29]. In patients with preserved LVEF, assessment of late gadolinium enhancement (LGE) presence and distribution patterns on cardiac MRI might improve patient risk stratification [30,31].

## 5. Description of Diagnostic Methods

### 5.1. Biomarkers

The identification of a biomarker of myocarditis remains a challenge because of the multifactorial characteristic of the disease, making it a very heterogenous entity. Current biomarkers used to detect myocardial injury are not disease specific, as they can be detected in most cardiac conditions [32]. Cardiac troponin is considered the most sensitive marker of myocardial injury. However, it is released in the highest concentrations during the acute phase of the disease and its sensitivity decreases significantly with time, which poses a major problem in the setting of chronic inflammation [33]. Additionally, normal troponin levels do not exclude myocarditis [3]. However, increased troponin in the absence of a known non-inflammatory cause in addition to abnormalities in other diagnostic tests increase the probability of myocarditis. 

N-terminal pro-B-type natriuretic peptide (NT-proBNP) may be indicative of HF regardless of its etiology, thus it has low specificity. High-sensitivity C-reactive protein, markers of systemic immune disease and specific tests for infective pathogens (i.e., SARS-CoV-2, Borrelia, cytomegalovirus, Epstein–Barr virus, human immunodeficiency virus, etc.) are of very limited value and therefore are not recommended [34]. 

An elevated eosinophil blood count may indicate underlying disorders, i.e., parasitic infection, allergy, drug- or vaccine-related hypersensitivity reaction, myeloproliferative disease, or an idiopathic hypereosinophilic syndrome [3]. However, isolated idiopathic eosinophilic forms with or without peripheral eosinophilia exist. Thus, once again, EMB is key to identifying specific myocarditis histotypes with a dismal prognosis [3].

There is an increasing need for a biomarker that is specific to the inflammatory process and fibrosis, and, preferably, correlates with the severity of the disease and serves as a prognostic factor. Existing data show that elevated serum levels of soluble ST2 (an inflammatory biomarker) can predict an increased risk of HF, but the diagnostic potential of soluble ST2 in myocarditis has not been established so far [35,36]. 

### 5.2. Serum Anti-Heart Autoantibodies

Circulating anti-heart autoantibodies (AHAs) are found in up to 60% of patients with myocarditis and inflammatory cardiomyopathy and 30% of their at-risk symptom-free relatives [8]. They are cardiac and disease specific, being detectable in only 1% of individuals with cardiac diseases excluding myocarditis/inflammatory cardiomyopathy and in 3% of healthy subjects [37]. They are detectable early in the course of the disease, even years before the onset of symptoms [38], which allows for their application in screening for myocarditis. They recognize multiple cardiac antigens, particularly myosin [39]. Autoantibodies with distinct autoantigen specificities may have a direct pathogenic or prognostic role in immune-mediated cardiomyopathy [3,37].

Organ- and disease-specific serum AHA or anti-intercalated disk autoantibodies (AIDA) suggest isolated autoimmune or immune-mediated myocarditis in the context of SIDs [3,40,41]. The measurement of serum AHA and/or AIDA of IgG class should be utilized to identify patients that can especially benefit from immunosuppression [14]. As some of AHAs may have a direct pathophysiological effect, their level may also serve as a prognostic factor. They are associated with diminished cardiac function, and with poor improvement in LVEF and increased diastolic stiffness at the 6-month control examination in patients with myocarditis, which may be attributable to enhanced myocardial fibrosis [42]. AHAs have also been described as markers of the autoimmune process in arrhythmogenic right ventricular cardiomyopathy probands and at-risk relatives [43].

### 5.3. Micro-RNA

MicroRNAs (miRNAs; endogenous single-stranded non-coding RNA) are pivotal regulators (enhance or suppress translation) of heart function, influencing cardiac differentiation, proliferation, apoptosis, myocardial injury, and inflammation [8,44]. 

Two categories of miRNAs can be distinguished: intracellular miRNAs identified in heart biopsies and circulating miRNAs detectable in body fluids (i.e., blood). The first category encompasses miRNAs that are present and detectable in cardiac tissue. The potential role of tissue miRNAs in myocarditis and inflammatory cardiomyopathy has been investigated in several studies; however, the infrequent performance of EMB means this method is unavailable for broader use in clinical practice [45,46]. Circulating miRNAs are considered promising biomarkers, as they are stable in body fluids and resistant to degradation by endogenous RNAses [47]. Their increase is observed in the course of myocarditis and it has even been proven that they correlate with disease severity and have prognostic value [48]. A novel miRNA has been described to differentiate myocarditis with or without pseudo-infarct presentation from acute myocardial infarction [49].

However, there is a need for bigger well-designed studies on biopsy-proven patients, as the results available at the moment are conflicting, with rather low reproducibility [50]. MiRNAs might serve as sensitive biomarkers and distinguish specific myocarditis etiologies. 

### 5.4. Imaging

The availability of a wide range of non-invasive imaging modalities allows for integrated patient evaluation and decisions regarding further treatment and disease monitoring [3,5].

#### 5.4.1. Echocardiography

Standard transthoracic echocardiography (TTE) should always be performed as the initial diagnostic work-up in all patients with suspected myocarditis/inflammatory cardiomyopathy. It allows rapid and portable acquisition, provides crucial information on cardiac morphology and function, and helps in differential diagnosis [3,8,51,52]. Early use of echocardiography is highly recommended as it allows assessment of the severity of cardiac compromise and potential complications related to myocarditis (i.e., thrombus, wall rupture) [51]. In patients with fulminant myocarditis, TTE may select patients directly for EMB without delay for CMR [16]. Nevertheless, standard echocardiography has some limitations in the evaluation of myocardial performance. 

Speckle tracking echocardiography (STE) is a promising ultrasound technique used for assessing myocardial function [53,54]. This method analyzes the motion of characteristic speckle patterns (natural myocardial acoustic markers) during the cardiac cycle. It allows for offline calculation of myocardial velocities and intrinsic cardiac deformation (strain and strain rate). Strain parameters are considered to be more sensitive and reproducible than conventional parameters in the detection of subclinical myocardial dysfunction [55]. STE should become part of the routine clinical practice in any patient with suspected acute myocarditis. STE is particularly recommended for patients with preserved LVEF [31], suspected chemotherapy-related cardiotoxicity [56], and cardiac involvement in the course of amyloidosis or sarcoidosis [57]. Moreover, there is also evidence that STE strain may be substituted for CMR LGE imaging [55]. The echocardiographic parameters have also predictive value, i.e., significantly impaired global longitudinal strain rate and global longitudinal strain are correlated with adverse cardiovascular events [58,59]. Although this method requires specific software, and depends on a higher image quality, it should be implemented in patients’ follow-up. 

#### 5.4.2. Cardiac Magnetic Resonance

The unique possibility of myocardial tissue characterization and very high-resolution anatomical and functional imaging, inter-observer consistency, and safety (CMR does not use ionizing radiation or iodine contrast agents) makes CMR the non-invasive gold-standard method for the diagnosis of suspected myocarditis in both acute and chronic settings [3,8,51,60]. Moreover, CMR can provide prognostic information and may help in patients’ follow-up and in assessing response to treatment [8,51].

The acute phase of inflammation initiates local or global cell injury and immune response, with inflammatory cell infiltration causing myocyte swelling and fluid accumulation in the interstitial space, resulting in edema, hyperemia with capillary leakage, and eventually necrosis. In severe cases, the prolonged inflammatory process leads to the replacement of altered myocardial regions by collagen with the formation of interstitial fibrosis and scars that can progress to dilated cardiomyopathy [8,60].

The Lake Louise Criteria (LLC) are the recommended diagnostic CMR criteria for patients with clinically suspected myocarditis. The original LLC allows diagnosis of myocardial inflammation when at least two of three tissue-based CMR markers are present: (1) edema (visible on T2-weighted imaging as increased signal intensity of the myocardium, prior to intravenous administration of a gadolinium-based contrast agent), (2) hyperemia/capillary leakage (increased regional uptake of the gadolinium contrast agent by abnormal myocardium during the first minutes after injection; early gadolinium enhancement, EGE), and (3) fibrosis/necrosis (visualized using the late gadolinium enhancement (LGE) technique (≥10 min after contrast agent injection)) [2,3]. The use of gadolinium contrast agents helps to differentiate non-ischemic cardiomyopathies (usually with mid-wall to subepicardial layer involvement without correspondence with any particular coronary artery distribution) and ischemic cardiomyopathies (subendocardial or transmural distribution) and detect different types (acute/chronic/healed) of injuries that occur during myocarditis; however, there are some limitations. Importantly, the diagnostic accuracy of LLC significantly decreases for patients with chronic symptoms [51,60]. In cases with an acute onset, myocardial edema in the absence of LGE has been associated with reversible myocardial injury and improved outcomes [61]. In contrast, LGE may accumulate in the expanded extracellular space of tissue swelling from myocardial edema; thus, it does not necessarily indicate irreversible myocardial injury and may be insensitive when distinguishing recent from remote myocarditis [3,51].

Recently, newer parametric imaging techniques (particularly T1 and T2 mapping and extracellular volume (ECV)) have been developed, which may overcome the limitations of LLC, and show promise in helping clinicians in their clinical management of a wide range of cardiac diseases [51]. According to the revised 2018 LLC, acute myocardial inflammation may be diagnosed if at least one specific CMR marker for edema is present (T2-weighted images or T2 mapping) with at least one additional T1-based marker for associated myocardial injury (LGE, T1 mapping, or ECV) [51]. T2 mapping was shown to be able to discriminate acute/active from healed myocarditis [62]. T1 mapping is especially useful for ruling out myocardial inflammation with a high negative predictive value of 92% [63]. In contrast to conventional CMR techniques, CMR parametric mapping provides direct quantitative (pixel-by-pixel) comparisons inter- and within-individuals of the magnetic properties of tissue, typically referred to as the relaxation times T1 and T2. Moreover, new CMR techniques allow the identification of pathologic processes without the need for contrast agents [51]. However, there are various limitations, which hinter the integration of this method into clinical routine: the lack of reference T1 and T2 values in myocarditis, the lack of expertise in the new CMR technique, and, last but not least, the lack of extensive correlations against the gold standard, i.e., biopsy-proven myocarditis, since most accuracy figures are based on clinically suspected disease [8].

#### 5.4.3. Nuclear Medicine

Positron emission tomography (PET) is another emerging imaging tool with the potential to provide complementary information about the inflammatory process in the myocardium [64,65]. Several tracers have been tested to detect enhanced myocardial metabolism and thus underlying inflammation [66]. The most commonly used, 18F-Fluorodeoxyglucose (18F-FDG), which is taken up by cells with increased glucose metabolism, has an established role in the diagnosis of sarcoidosis with cardiac involvement and therapy monitoring [67,68]. 18F-FDG uptake closely matches LGE and myocardial edema detected by CMR [69,70,71]. 

The combination of PET-MRI imaging might allow considerable improvement in diagnostic accuracy, especially in cases with ambiguous MRI findings and myocarditis with HF or arrhythmia presentation when the accuracy of CMR is limited [72]. In selected patients, particularly those with non-compromised cardiac function on echocardiography or with myocarditis with HF or arrhythmia presentation, this approach may facilitate the decision for EMB [60,70,71]. CMR and PET assess the entire heart and therefore add additional information about the extent of the inflammatory process in the myocardium, which may be important in risk stratification. Additionally, the advantage of PET is the possibility of using it in patients with artificial prosthetic heart valves and with cardiac implantable devices, for whom CMR cannot be used [73]. However, there are several key limitations: poor imaging of the right ventricle, exposure to radiation, imaging time, relatively low accessibility, and high cost of the study. 

### 5.5. Endomyocardial Biopsy: Diagnostic Gold Standard

Since the pathophysiological changes of myocarditis occur at cellular and subcellular levels, imaging technologies cannot replace EMB in the diagnosis of myocarditis. The role of EMB in the diagnosis of unexplained cardiomyopathy/suspected myocarditis is invaluable. EMB is still a diagnostic gold standard allowing establishment of the etiology (i.e., virus positive/negative, immune-mediated myocarditis, sarcoidosis), definition of the type of inflammatory process (active, chronic, or healed), and degree of myocardial fibrosis, and is useful in differential diagnosis (i.e., amyloidosis, infiltrative/storage disease, arrhythmogenic cardiomyopathy) [3,74]. Current immunohistochemical and molecular analyses are accurate and allow for characterization and quantification of the inflammatory infiltrates (lymphocytic, eosinophilic, giant-cell, sarcoid) of myocardial fibrosis and/or viral infection [3,75]. According to recommendations, EMB should be considered in all patients with clinically suspected myocarditis that is defined according to the ESC 2013 criteria to reach a certain etiological diagnosis [3,34] and plan etiology-directed therapy [34,76].

EMB is crucial in establishing the diagnosis of patients with suspected complicated acute/fulminant myocarditis frequently associated with acute HF and life-threatening arrhythmias/heart block. Especially, suspicion of giant-cell/eosinophilic myocarditis requires a rapid EMB to be performed to start specific treatment. In these situations, CMR should not delay EMB. On the other hand, EMB should be considered in patients with unexplained chronic cardiomyopathy that is progressive or unresponsive to standard therapy, as, especially in these patients, the accuracy of non-invasive imaging modalities (TTE, CMR) is low [72,76].

The accuracy of EMB may be increased with biventricular sampling, collection of 5–10 tissue samples from different heart regions (RV and/or LV), broad immunohistochemical and biomolecular analyses (polymerase chain reaction (PCR), specific miRNA), and CMR-, PET-, or electroanatomic-guided EMB according to the distribution of inflammatory regions (in relation to LGE, 18F-FDG, or disturbed electric potential localization, respectively) [77,78,79,80]. EMB may also be repeated when there is suspicion of sampling error.

The safety of EMB is an issue often raised by skeptics of the procedure, but it should be emphasized that especially in experienced centers, the risk of complications is very low (0–0.8%) [75,76,81]. The most common adverse effects are vascular complications, but these may be almost completely reduced with careful choice of the access site and use of an imaging guide (i.e., ultrasound, fluoroscopy). 

## 6. Diagnosis and Decision-Making Process

**Figure 2:**
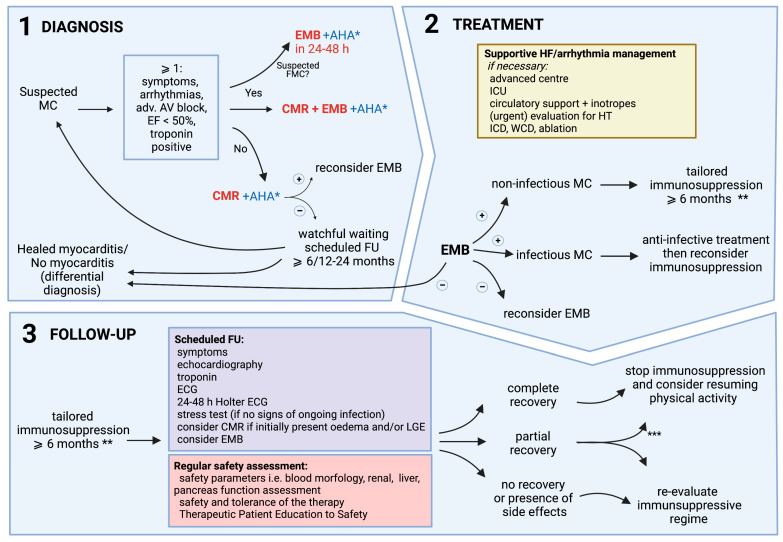
Schematic depiction of a diagnosis, decision-making process, and individualized treatment approach for patients with myocarditis. * if available (positive AHA additionally supports the implementation of immunosuppression); ** i.e., type of myocarditis (lymphocytic, eosinophilic, giant-cell), AHA, contraindications (see Table 4 for safety assessments); *** low probability of further improvement, no signs of active inflammation; (+): positive result for myocarditis; (−): negative result for myocarditis; AHA: anti-heart autoantibodies; AV: atrioventricular; CMR: cardiac magnetic resonance; ECG: electrocardiogram; EF: ejection fraction; EMB: endomyocardial biopsy; FMC: fulminant myocarditis; FU: follow-up; HF: heart failure; HT: heart transplant; ICD: implantable cardioverter defibrillator; ICU: intensive care unit; LGE: late gadolinium enhancement; MC: myocarditis; WCD: wearable cardioverter defibrillator. Created with BioRender.com (accessed on 4 January 2022).

The management of myocarditis is complex and should be multidisciplinary, including a cardiologist, radiologist, cardiovascular pathologist, clinical immunologist, or rheumatologist and an infectious disease specialist [34,76]. Due to the variability in its clinical presentation, the diagnosis of myocarditis is frequently challenging. Thus, a uniform approach to the diagnostic process is recommended. The identification of patients with suspected myocarditis is based on a non-invasive work-up in keeping with the ESC 2013 criteria for clinically suspected myocarditis (Table 2) [3]. The more criteria are fulfilled, the higher the likelihood of myocarditis. In order to confirm the diagnosis and provide a safe and disease-specific treatment, EMB should be performed without delay, particularly in patients with high-risk features at presentation. Myocardial samples should then undergo complex immunohistochemical and molecular evaluation [3,34]. The proposed diagnostic and decision-making process is presented in Figure 2. 

## 7. Personalized Treatment, Follow-Up, and Return to Activity

### 7.1. Personalized Treatment

Treatment of myocarditis should include two elements: the optimal care of HF and arrhythmias in accordance with current guidelines (regardless of its etiology), and the involvement of disease-specific therapies based on EMB (and AHA if available) results [14]. Both strategies should be adjusted to the severity of the clinical profile and the occurrence of a short-term spontaneous or treatment-induced recovery. Disease-specific (personalized) treatment should always be considered, as standard cardiovascular therapy can only delay the progression of the disease to dilated cardiomyopathy (Table 3).

In fulminant myocarditis with hemodynamic instability, patients should be referred to tertiary centers due to the possible necessity for cardio-pulmonary support. 

For infectious-negative forms of acute and chronic myocarditis with EMB-proven inflammation (particularly those with the presence of AHA), immunosuppressive treatment should always be considered [3,14,34]. Current data support immunosuppression (with other guideline-recommended HF medications) in giant-cell and eosinophilic myocarditis and cardiac sarcoidosis [34]. 

Some single-center studies (i.e., Wojnicz et al. and Frustaci et al) and meta-analysis also reported a beneficial effect of immunosuppressive therapy in lymphocytic myocarditis with chronic HF presentation [9,82,83]. Currently, a multicenter randomized study (IMPROVE-MC EudraCT: 2020-003877-23) on combined prednisone and azathioprine immunosuppression in virus-negative myocarditis is ongoing [84]. Although more clinical data is required in relation to the timing and the length of immunosuppression, it is worth noting that the response to immunosuppression is a major diagnostic criteria of autoimmune disease; therefore, immunosuppression is, by definition, warranted in biopsy-proven infectious-negative myocarditis and it is unethical not to treat patients with ventricular dysfunction and/or arrhythmia refractory to standard cardiological treatment [85]. 

To date, no specific recommendations have been provided for the use of antiviral therapies and intravenous immunoglobulins in myocarditis because of a lack of controlled studies; therefore, such therapies are currently off-label and should be decided using a personalized approach following consultation with infectious disease specialists [3,74].

### 7.2. Tailored Therapy and Follow-Up

Immunosuppressive treatment should be started, especially in the presence of LV (or isolated RV) systolic dysfunction and/or persistent and severe arrhythmia [15,34]. It should be continued for at least 6–12 months. Treatment should be targeted at the lowest level of disease activity. Furthermore, current evidence seems to support more intensive and prolonged immunosuppressive treatment in patients with systemic inflammatory/autoimmune diseases and infectious-negative autoimmune/immune-mediated myocarditis.

Before starting and during immunosuppressive treatment in biopsy-proven virus-negative myocarditis, a safety checklist should be assessed routinely to rule out contraindications to the therapy (Table 4) [86,87]. 

A prospectively scheduled close follow-up (at least 6–24 months) after diagnosis of myocarditis is of great importance in prognostic stratification and disease management. The duration of immunosuppressive therapy should be tailored to the patient, as immediate discontinuation of immunosuppressive drug administration may be frequently followed by prompt disease remission (particularly in giant-cell/eosinophilic myocarditis and cardiac sarcoidosis) [74]. The specific moment when the therapy should be withdrawn after LVEF recovery is not well defined and should be adjusted individually. Follow-up evaluation should be based on clinical (sign and symptoms of HF, arrhythmia, and/or chest pain), biochemical (troponin), electrocardiographic, and echocardiographic assessment. Additional tests (Holter-ECG, exercise test, CMR) may also be useful (see below the return to physical activity). Although CMR may be useful, routine CMR for follow-up disease monitoring is not recommended. A control EMB may also be considered to guide the length of the therapy.

Patients with myocarditis should also be adequately educated about the natural course of the disease, the need for patients to adhere to the recommended therapy, and physical activity restrictions. Possible adverse effects of immunosuppressive treatment should also be discussed with the patient to avoid the risk of poor compliance. 

### 7.3. Physical Activity

Current ESC guidelines recommend a more flexible approach regarding the avoidance of physical activity, reflecting a tendency to adapt prescription to the individual [88]. It has been clearly stated that patients with a definitive (EMB-based) or a probable (clinician-based) diagnosis of myocarditis are discouraged from participating in both competitive and leisure sports if active inflammation is present.

The duration of obligatory restriction from exercise programs for competitive or recreational sports with moderate and high intensities is set at a minimum of 3–6 months, depending on the clinically assessed severity, progression/regression of the disease, duration of illness, LV function at onset, and extent of inflammation on the CMR, which emphasizes the superiority of general assessment supported by imaging in fixed time frames. Comprehensive evaluation should include both clinical assessment and diagnostic procedures. Factors influencing the resumption of training are presented in Table 5. 

Return to exercise (even competitive sports) should be considered after 3–6 months in patients with presumed or biopsy-proven healed myocarditis, with no symptoms, in the absence of myocardial oedema and persisting LGE areas in CMR at 3–6 months (Table 6). Individuals with large areas of LGE (>20%) and abnormal LV function should be discouraged from participating in physical activity of a moderate to high intensity. Patients with previous myocarditis are at increased risk for recurrence or silent clinical progression of the disease; therefore, a periodical annual re-assessment (particularly in patients with the presence of LGE at baseline) is recommended.

## Figures and Tables

**Table 1:** Important definitions required for accurate diagnosis of myocarditis.

Definite Diagnosis of Myocarditis Based on Endomyocardial Biopsy	
**Myocarditis**	EMB-proven myocarditis confirmed by histological and immunohistochemical criteria and presence of abnormal inflammatory infiltrate: - typically ≥14 leucocytes/mm2 including up to 4 monocytes/mm2, with the presence of CD3-positive T lymphocytes ≥ 7 cells/mm2; - specific cells, i.e., eosinophils, giant-cell, sarcoid granulomas; Additional analyses (i.e., molecular) necessary for etiology assessment; ±serum positive AHA
**Infective myocarditis**	EMB-proven myocarditis confirmed by histological and immunohistochemical criteria; Specific infective agent detected in EMB; - i.e., EMB viral PCR positive, borrelia (Lyme disease) positive; ±serum AHA positive
**Myocarditis temporarily associated with infective agent**	Possible or proven systemic infection (i.e., positive nasal swab for virus); EMB for infective cause negative
**Immune-mediated myocarditis**	EMB-proven myocarditis confirmed by histological and immunohistochemical criteria; ±systemic immune-mediated diseases (lupus erythematosus, GPA); EMB for infective cause typically negative; ±serum positive AHA
**Autoimmune myocarditis**	Organ-specific autoimmune myocarditis, exclusion of other known inflammatory causes; EMB-proven myocarditis confirmed by histological and immunohistochemical criteria; EMB for infective cause typically negative; ±serum positive AHA
**Clinically suspected myocarditis**	Suspicion of myocarditis based on clinical presentation and non-invasive tests (according to ESC criteria [3,5]); Without EMB confirmation
**Inflammatory cardiomyopathy**	Biopsy-proven myocarditis with systolic and/or diastolic cardiac dysfunction

AHA: anti-heart autoantibodies; CD: cluster of differentiation; EMB: endomyocardial biopsy; ESC: European Society of Cardiology; GPA: granulomatosis with polyangiitis; PCR: polymerase chain reaction; ±: with or without.

**Table 2:** Recommended diagnostic tests for the diagnosis of myocarditis.

Clinical Presentation and Diagnostic Tests	Method/Characteristic	Recommendation		
		Acute Cardiac Signs/Symptoms	Chronic Cardiac Signs/Symptoms	Follow-Up
**Diagnosis of clinically suspected myocarditis**: new unexplained signs/symptoms and ≥1 non-invasive test being positive from diagnostic categories (ECG, troponin(s), cardiac imaging (echo/CMR), tissue characterization), if patient is asymptomatic ≥2 positive non-invasive tests [3] *				
Evaluation of medical history and physical examination	Low sensitivity and specificity. Mandatory evaluation for: Clinical presentation: ischemic, HF, cardiogenic shock, arrhythmic; Symptoms: chest pain, dyspnea, palpitations, syncope, etc. and time of onset; Medical history: suspected SID, previous clinically suspected or confirmed myocarditis (including family history), toxic agents; Preceded respiratory or gastrointestinal infection.	++	++	++
Coronary angiography (invasive or CT)	Mandatory for exclusion of: Significant changes in coronary arteries (CT preferred in patients with low pretest probability of CAD).	++	++	-
Laboratory evaluation	Intermediate sensitivity and low specificity. Mandatory evaluation for: Troponin increase; NTproBNP increase indicative for HF; (Other biomarkers low sensitivity and low specificity) Valuable for follow-up.	++	++	++
-AHA	Intermediate sensitivity and intermediate specificity. Evaluation valuable for: AHA indicate immune-mediated forms (particularly benefits from immunosuppression). Valuable for follow-up.	++	++	++
ECG	High sensitivity and low specificity. Mandatory evaluation for: Conduction abnormalities; PR segment depression or elevation; ST-T wave change (ST segment elevation or non-ST elevation, T wave inversion); Atrial or ventricular arrhythmias; Reduced R wave height, abnormal Q waves, low voltage. Valuable for follow-up.	++	++	++
Echocardiography	High sensitivity and low specificity. Mandatory evaluation for: Regional wall motion or global systolic or diastolic abnormalities; Chambers dilation; Increased wall thickness, Pericardial effusion; Endocavitary thrombi or other acute complications. Valuable for follow-up.	++	++	++
CMR	High sensitivity and intermediate specificity **. Mandatory evaluation for ***: Complementary information on cardiac morphology and function (see echocardiography above; particularly useful when echocardiography is inconclusive); Tissue characterization: edema, inflammation and fibrosis detection, quantification and localization through T1 and T2 mapping, extracellular volume assessment and LGE (updated LLC 2018 criteria). Valuable for follow-up (especially in patients with persistent dysfunction at echocardiography, arrythmias, or ECG abnormalities)	++	++	++
PET-CT/MR	May be useful when: Contraindication to CMR/CMR was inconclusive (particularly in chronic cardiac signs/symptoms); Suspected SID, especially cardiac sarcoidosis. May be used for follow-up.	(+)	(+)	(+)
**Confirmation of myocarditis**: clinically suspected myocarditis + EMB				
EMB	High-intermediate sensitivity and high specificity. Mandatory evaluation for: Histology; Immunohistochemistry (anti-CD3-, CD4-, CD8-, CD68-, HLA-ABC, HLA-DR); Molecular and other analyses/stains if necessary. Recommended in all patients (particularly when myocardial compromise, progressive or persistent severe cardiac dysfunction and/or life-threatening ventricular arrhythmias and/or advanced AV block with lack of short-term (<1–2 weeks) expected response to usual medical treatment) in order to establish diagnosis and allow for disease-specific therapy. May be used for follow-up.	++	++	++

‘−’: not recommended; ‘(+)’: may be considered; ‘++’: should be considered. * in the absence of other conditions (i.e., significant valvular heart defects, congenital heart disease, stress-induced cardiomyopathy, thyroid disease) that could be responsible for the clinical presentation; ** sensitivity and specificity may be significantly decreased in chronic inflammatory cardiomyopathy, particularly in sub-clinical forms; *** CMR should be performed in all patients with clinically suspected myocarditis and significant CAD excluded or unlikely. AHA: anti-heart autoantibodies; AV: atrioventricular; CAD: coronary artery disease; CMR: cardiac magnetic resonance; ECG: electrocardiogram; CD: cluster of differentiation; CT: computed tomography; EMB: endomyocardial biopsy; HF: heart failure; HLA-ABC: human leukocyte antigen-ABC; HLA-DR: human leukocyte antigen-DR; LGE: late gadolinium enhancement; LLC: the Lake-Louise criteria; NTproBNP: NT-proB-type natriuretic peptide; PET: positron emission tomography; SID: systemic immune-mediated disease.

**Table 3:** Personalized treatment regimens for patients with myocarditis/inflammatory cardiomyopathy.

Treatment	Recommendation
**Standard and/or supportive treatment**	
Standard HF medications (ACE-I/ARNI, beta-blocker, MRA, ivabradine, SGLT2-I, diuretic, etc.)	Management according to the current appropriate guidelines.
Therapy of end-stage or acute HF with hemodynamic compromise	Treatment in experienced intensive (cardiac) care unit. Advanced cardio-pulmonary support may be needed as a bridge to heart transplantation or recovery. If possible, referral for a heart transplant/LV assist device implantation should be deferred for at least 3–6 months.
Standard antiarrhythmic medications (i.e., amiodarone)	The management of arrhythmias should mainly be supportive, as in myocarditis, arrhythmias often diminish or disappear following the resolution of acute myocardial inflammation. Patients with life-threatening arrhythmias should be referred to experienced centers.
Nonsteroidal anti-inflammatory drugs (i.e., ibuprofen) and colchicine	Patients with mild myocarditis and predominant associated pericarditis (pericarditic chest pain, pericardial effusion, high C-reactive protein) with preserved or nearly preserved LV function. Potentially harmful in other groups, but data is lacking.
Anticoagulation	Patients with acute/fulminant myocarditis with reduced LVEF until resolution of the acute inflammatory phase may require anticoagulation. Patients with intracardiac thrombosis and peripheral embolization, particularly if biopsy-proven eosinophilic myocarditis.
Catheter ablation	No indication in acute myocarditis. If necessary, it may be considered in selected patients with drug-refractory or scar-related arrhythmias or arrhythmic storms (i.e., in giant cell myocarditis).
ICD/CRT	Indications for ICD/CRT implantation should be evaluated individually; however, urgent ICD implantation in primary SCD prevention is not recommended for patients with recent-onset myocarditis. The decision regarding ICD/CRT implantation should be deferred for at least 3–6 months. A wearable cardioverter defibrillator can provide protection as a bridge to ICD or transplant decision, or to recovery after immunosuppressive therapy, particularly in patients with high arrhythmic risk and/or severe left ventricular dysfunction.
**Disease-specific treatment**	
Withdrawal of potential triggering factors (i.e., clozapine, immune-checkpoint inhibitors)	Myocardial damage induced by toxic substances or drugs may progress if treatment is not stopped immediately.
Anti-infectious treatment	Therapy (anti-viral, antibiotics, antifungal, antiparasitic) directed against specific infectious agents (i.e., HIV, HHV6, Parvovirus B19, Borrelia).
Immunosuppressive *treatment* in specific infectious-negative forms	Recommended for immune-mediated forms confirmed with EMB (and AHA if available). Giant-cell myocarditis: triple therapy with steroids, cyclosporin, azathioprine; Eosinophilic myocarditis: dual therapy with steroids and steroid-sparing drug (azathioprine, cyclosporine, or mycophenolate mofetil as alternatives); Cardiac sarcoidosis: dual therapy with steroids and steroid-sparing drug (azathioprine, cyclosporine, or mycophenolate mofetil as alternatives); Lymphocytic myocarditis: most commonly prednisone (starting from 1 mg/kg for 1 month and maintenance of 0.33 mg/kg for 5 months) with azathioprine (2 mg/kg for at least 6 months); cyclosporine or mycophenolate mofetil as alternatives; Specific disease-directed therapy (i.e., rituximab, methotrexate) if myocarditis occurs in the context of systemic inflammatory/autoimmune disease (i.e., GPA, lupus erythematosus)

ACE-I: angiotensin-converting enzyme inhibitors; ARNI: angiotensin receptor neprilysin inhibitor; AHA: anti-heart autoantibodies; CRT: cardiac resynchronization therapy; EMB: endomyocardial biopsy; HF: heart failure; HIV: human immunodeficiency virus; HHV6: human herpesvirus 6; GPA: granulomatosis with polyangiitis; ICD: implantable cardioverter defibrillator; LV: left ventricle; LVEF: left ventricle ejection fraction; MRA: mineralocorticoid receptor antagonists; SGLT2-I: sodium-glucose co-transporter-2; SCD: sudden cardiac death.

**Table 4:** Safety checklist used before starting and during immunosuppressive treatment.

Use of the safety checklist is intended to rule out potential general and individual risks related to immunosuppressive therapy.
**Before starting** Patients who are candidates to immunosuppressive therapy should be screened for:
Common latent infections (i.e., HBV, HCV, HIV, EBV, CMV, borreliosis, tuberculosis); Hidden malignancy (i.e., in situ prostatic, cervical, paraproteins and other chronic hematological malignancy, particularly patients aged ≥40 years old; TPMT deficiency or mutation in candidates for azathioprine treatment (patients with reduced TPMT activity following the administration of thiopurines are at greater risk of adverse drug reactions, even with low-dose azathioprine treatment); Therapeutic Patient Education to Safety: patients (and/or caregivers) must be educated to self-manage immunosuppressive therapy and about risks related to the disease and prescribed treatment;
**During treatment**
At all follow-up visits, the patient should be asked about the symptoms of possible infection, HF symptoms, signs of hepatic, renal and/or pancreatic injury, and (pre-)diabetes; At all follow-up visits, the patient should be educated about healthy lifestyle and restrictions (i.e., diet, physical activity, prevention of infectious complications, contraception); At all follow-up visits, concomitant medications should be verified for restricted therapies (i.e., allopurinol on treatment with azathioprine);

CMV: cytomegalovirus; EBV: Epstein–Barr virus; HBV: hepatitis-B virus; HCV: hepatitis-C virus; HF: heart failure; HIV: human immunodeficiency virus; TPMT: thiopurine methyltransferase.

**Table 5:** Factors affecting the resumption of training (based on [88,89]).

Factors Affecting the Resumption of Training
Relief of symptoms
Normalization of LV systolic function on echocardiography and CMR
Normal troponin and biomarkers of inflammation
Absence of: Active inflammation or LGE on CMR Clinically relevant arrhythmias during exercise on prolonged ECG monitoring
Good clinical status and functional capacity

CMR: cardiac magnetic resonance; ECG: electrocardiogram; LGE: late gadolinium enhancement; LV: left ventricle.

**Table 6:** Recommended tests for the follow-up assessment of patients with myocarditis (based on [88,89]).

Patient Group	Individuals with Presumed or Biopsy-Proven Healed Myocarditis
**Aim of the test**	For routine control evaluation, in order to assess the risk of exercise-related SCD
**Recommended modalities**	Measurement of troponin and biomarkers of inflammation General assessment by echocardiography Prolonged ECG monitoring (48 h) Exercise stress test (patients without symptoms and known ongoing inflammation) CMR (in case of presence of myocardial oedema/LGE areas at the acute phase of the disease)
**When/how often**	At 3–6 months after the acute phase of the disease and then annually

CMR: cardiac magnetic resonance; ECG: electrocardiogram; LGE: late gadolinium enhancement; SCD: sudden cardiac death.
